# Mass spectrometry-based proteomics for advancing solid organ transplantation research

**DOI:** 10.3389/frtra.2023.1286881

**Published:** 2023-11-24

**Authors:** Che-Fan Huang, Pei Su, Troy D. Fisher, Josh Levitsky, Neil L. Kelleher, Eleonora Forte

**Affiliations:** ^1^ Proteomics Center of Excellence, Northwestern University, Evanston, IL, United States; ^2^ Department of Chemistry, Northwestern University, Evanston, IL, United States; ^3^ Division of Gastroenterology and Hepatology, Comprehensive Transplant Center Northwestern University Feinberg School of Medicine, Chicago, IL, United States; ^4^ Department of Biochemistry and Molecular Genetics, Northwestern University Feinberg School of Medicine, Chicago, IL, United States; ^5^ Department of Surgery, Feinberg School of Medicine, Comprehensive Transplant Center, Northwestern University, Chicago, IL, United States

**Keywords:** proteomics, proteoforms, bottom-up mass spectrometry, top-down mass spectrometry, biomarkers, solid organ transplantation

## Abstract

Scarcity of high-quality organs, suboptimal organ quality assessment, unsatisfactory pre-implantation procedures, and poor long-term organ and patient survival are the main challenges currently faced by the solid organ transplant (SOT) field. New biomarkers for assessing graft quality pre-implantation, detecting, and predicting graft injury, rejection, dysfunction, and survival are critical to provide clinicians with invaluable prediction tools and guidance for personalized patients' treatment. Additionally, new therapeutic targets are also needed to reduce injury and rejection and improve transplant outcomes. Proteins, which underlie phenotypes, are ideal candidate biomarkers of health and disease statuses and therapeutic targets. A protein can exist in different molecular forms, called proteoforms. As the function of a protein depends on its exact composition, proteoforms can offer a more accurate basis for connection to complex phenotypes than protein from which they derive. Mass spectrometry-based proteomics has been largely used in SOT research for identification of candidate biomarkers and therapeutic intervention targets by so-called “bottom-up” proteomics (BUP). However, such BUP approaches analyze small peptides in lieu of intact proteins and provide incomplete information on the exact molecular composition of the proteins of interest. In contrast, “Top-down” proteomics (TDP), which analyze intact proteins retaining proteoform-level information, have been only recently adopted in transplantation studies and already led to the identification of promising proteoforms as biomarkers for organ rejection and dysfunction. We anticipate that the use of top-down strategies in combination with new technological advancements in single-cell and spatial proteomics could drive future breakthroughs in biomarker and therapeutic target discovery in SOT.

## 1. Introduction

Solid organ transplantation (SOT) is the standard-of-care treatment for patients with end-stage organ disease (1). Despite considerable progresses made in the field to improve transplant outcomes, there are still significant challenges that must be overcome, including scarcity of high-quality organs suitable for transplant, suboptimal assessment of organ quality pre-transplantation, and unsatisfactory long-term organ and patient survival (2, 3).

**Figure 1:**
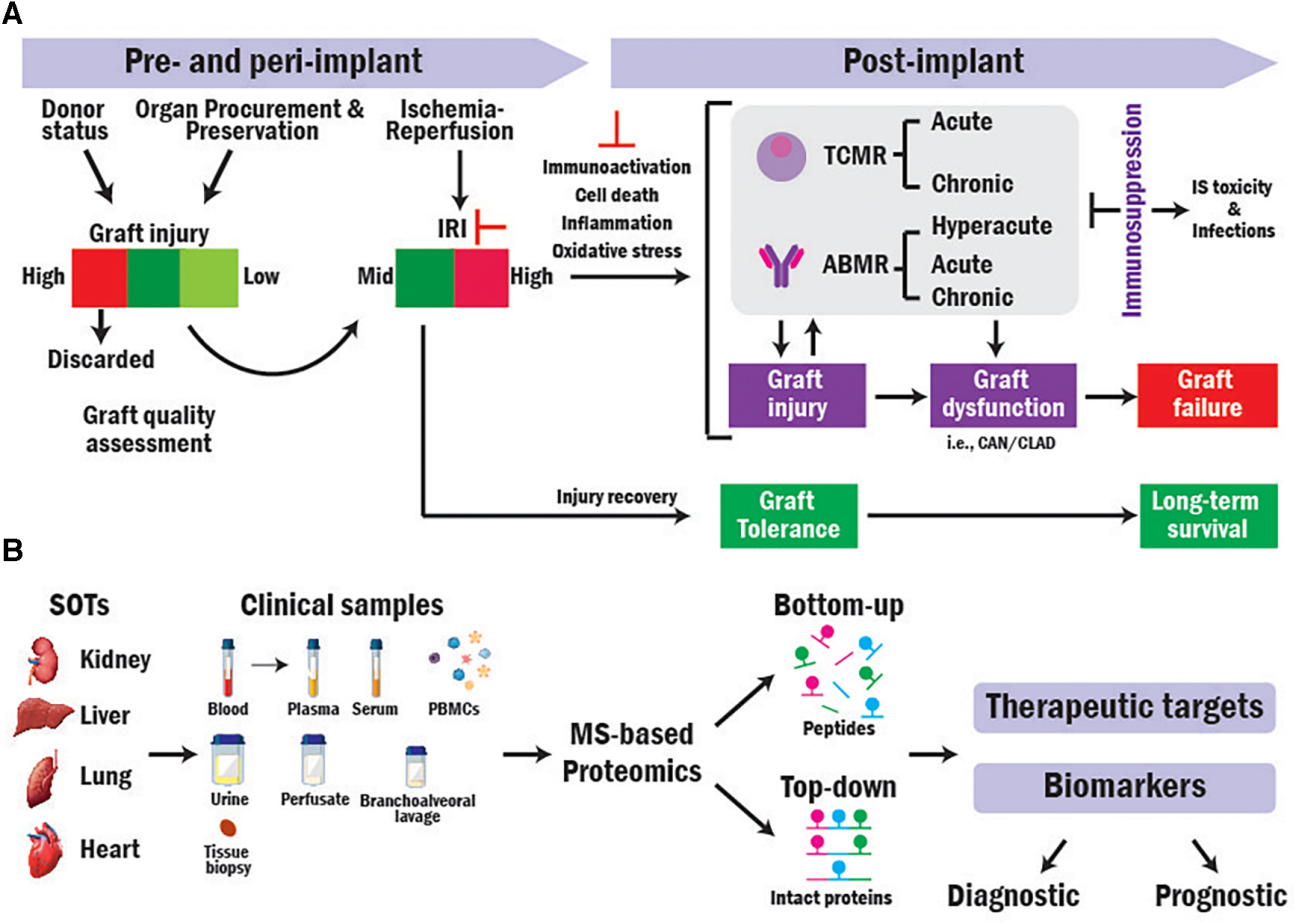
Translational MS-based proteomics for identifying novel biomarkers and therapeutic targets in SOT research. (**A**) Overview of mechanisms contributing to SOT outcomes. (**B**) Workflow of translational bottom-up and top-down MS-based proteomics approaches in SOT.

Most grafts derive from brain-dead (BD) donors. However, their usage results in higher rejection rates and worse transplant outcomes compared to those obtained from living donors that are less damaged in pre-implantation (4–6). Organs are subjected to damage from the pre- and peri-implantation procedures (i.e., Ischemia reperfusion injury, IRI) that can trigger higher recipient immune response (7, 8). Higher immunoactivation increases the risk of T-cell and antibody mediated rejections (TCMR and ABMR), graft dysfunction, and graft loss (9–12). While immunosuppressive (IS) therapies are used to prevent and treat rejection by dampening the recipient immune system, those cause toxicity and make the recipient more susceptible to infections, malignancies, and graft injury resulting in diminished graft survival (13) (Figure 1A).

Therefore, to increase the amount and quality of donor supply, avoid rejection, and improve long-term transplant outcomes, it is crucial to identify accurate and reliable biomarkers to (1) assess donor organ quality pre-implantation, (2) early detect immunoactivation and rejection, (3) monitor rejection and disease progression and the response of patients to immunotherapies. In addition, the transplant field lacks prognostic biomarkers that can predict (1) transplant outcomes before implantation based on organ quality, (2) graft rejection, dysfunction, and failure before their occurrence, and (3) the response of patients to immunosuppression. Such biomarkers could guide allocation, decision-making, patient risk stratification, and personalized treatments (14).

Mechanisms behind graft injury and rejection are not well understood. Their characterization is fundamental for developing new therapeutic strategies aimed to minimize injury due to graft origin, IRI and its sequelae to improve graft quality, function, and survival. More targeted graft pre-treatment would be particularly beneficial to expand the pool of organs suitable for transplant and improve long-term graft survival.

Proteins are directly responsible for the function, structure, and regulation of cells, tissues, and organs (15–17). Therefore, the identification and characterization of proteins involved in injury and rejection is crucial to advance transplant research.

Mass spectrometry (MS)-based proteomics can elucidate biological processes in depth (18, 19) and has been extensively used to analyze the protein composition of a variety of clinical samples from transplant recipients (20). There are two general approaches for performing MS-based proteomics analysis: Bottom-up (BUP) and Top-down proteomics (TDP). In BUP, proteins are digested into peptides prior to MS analysis, while in TDP, the analysis is conducted directly at intact protein level (Figure 1B) (21, 22). Here, we discuss the application of both approaches in SOT research.

## 2. Bottom-up proteomics in transplantation

**Figure 2:**
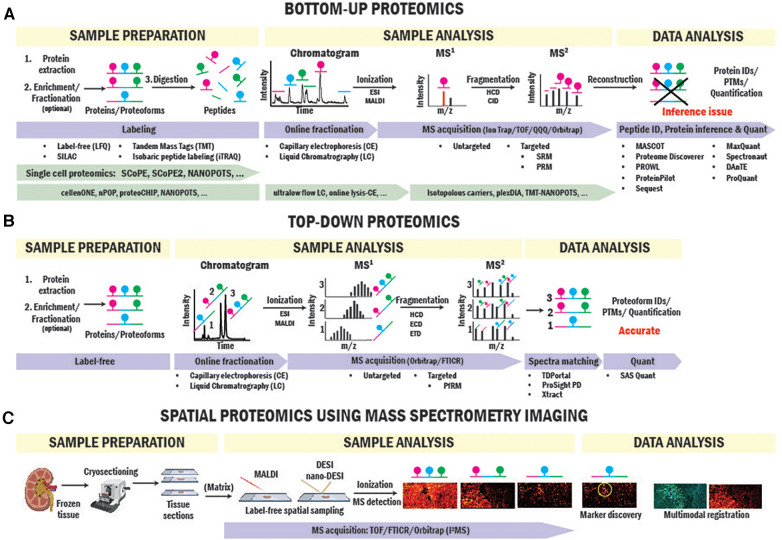
BUP and TDP strategies for biomarker and therapeutic target identification. Workflows of BUP (**A**), TDP (**B**) and spatial proteomics (**C**) using mass spectrometry imaging approaches.

BUP is the strategy traditionally used for proteomics studies (23–25). In BUP (Figure 2A), proteins are extracted from the clinical sample, enriched by immunoprecipitation or fractionation when needed, and digested with trypsin into peptides (26). Peptides are then seperated by liquid chromatography (LC) or capillary electrophoresis (CE), ionized by electrospray ionization (ESI) (27), and injected into the mass spectrometer for producing precursor ion (MS^1^) spectra. Next, individual peptides are fragmented via higher-energy collisional dissociation (HCD) or collision-induced dissociation (CID) to obtain the fragment ion (MS^2^) (28) spectrum in either untargeted or targeted mode. In untargeted mode, fragmentation is performed based on top-intensity peptides or MS^1^ mass-to-charge ratio (*m/z*)-selected ranges. In targeted mode, fragmentation targets are specific peptides with known sequence and *m/z* information. Data from MS^1^ and MS^2^ spectra is combined and used to sequence peptides, infer proteins, identify posttranslational modifications (PTMs), and quantify peptides/proteins present in the sample using a variety of computational engines (29–32). Aside from label-free quantitative approach that relies on the intensities of the peptides obtained in MS^1^ (33), labeling approaches are commonly used in BUP for relative and absolute quantitation. In particular, Tandem Mass Tags (TMT) and Isobaric tags (iTRAQ), use multiplex isobaric tags to label peptides and base the quantitation analysis on the intensity of reporter ions from the tag found in MS^2^ spectra (34, 35).

Most BUP studies aimed at identifying biomarkers to detect, monitor, and predict acute rejection, TCMR, ABMR, injury, and dysfunction while uncovering mechanisms behind injury and rejection to find novel therapeutic targets. Such studies have been conducted both on solid tissue biopsies (gold standard in the field but invasive), and the less invasive body fluids, including urine, blood, bronchoalveolar lavage (BAL) fluid, and perfusates. Importantly, the combined analysis of different sample types can help elucidating pathophysiological scenarios. For example, solid tissue biopsies can inform on the outset of the disease process (i.e., immune cell infiltration and allograft injury), while body fluids on the presence of circulating blood immunological proteins (i.e., antibodies) migrating into the site of injury (36) or proteins secreted from the allograft in the urine (37).

Kidney is the most transplanted organ (38). Given its physiological role, urine has been largely investigated for biomarker discovery (39). Sigdel et al. identified urine protein panels able to distinguish different conditions after kidney transplant, including acute rejection (AR), chronic allograft nephropathy, BK virus nephritis, and stable grafts (40). Other studies found potential urinary biomarkers for diagnosing acute tubulointerstitial renal allograft rejection (41) and chronic allograft nephropathy (i.e., beta-2-microglobulin) (42, 43); exosomal tetraspanin-1 and hemopexin for early detection of TCMR (44); collagen peptides and matrix metalloproteinase-8 as reporters for clinical and subclinical TCMR (45); matrix metalloproteinase-8 as indicator of renal allograft inflammation and injury (46); and a 10-protein panel distinguishing ABMR vs. no-ABMR (47).

BUP analysis of plasma, serum, and peripheral blood mononuclear cells (PBMCs) led to the identification of (1) several proteins with AR diagnostic value, including properdin, keratin 1, titin, kininogen-1, and lipopolysaccharide-binding protein (48, 49); (2) serum aminoacylase-1 as a potential diagnostic biomarker of delayed graft function (DGF) severity with moderate outcome predictive power (50); and (3) protein profiles with high predictive accuracies of Chronic Allograft Nephropathy severity (51).

BUP analysis of kidney biopsies identified signatures distinguishing healthy from injured tissues caused by TCMR or polyomavirus BK nephropathy (52); differentially expressed proteins in chronic rejection, including the alpha-1 chain of collagen type IV and Integrin alpha-1; and several prognostic candidates of fibrosis (53). Additionally, combining laser microdissection of glomeruli from biopsies with BUP has allowed for determining proteome profiles of active and chronic active ABMR and identified three potential ABMR biomarkers (54). Finally, various studies were conducted on tissue biopsies and perfusates of kidney from living or deceased donors after IRI, preservation, and DGF to elucidate injury mechanism and guide the development of improved organ preservation strategies to elevate graft quality (55–60).

Liver is the second most transplanted organ (38). Liver transplant BUP research mainly focused on mechanisms of IRI and acute rejection. Studies on biopsies showed that IRI alters proteins involved in lipid and energy metabolism, metabolic pathways, redox signaling, oxidative-stress response, and cytoskeleton remodeling and indicated Ras GTPase-activating-like protein IQGAP1 and Cytoplasmic protein NCK1 as potential targets to reduce IRI (61, 62).

The comparison of global proteomes of liver tissues from acute cellular rejection (ACR) and non-ACR transplant recipients showed that proteins in ACR were mainly involved in immune response and inflammation while those downregulated implicated in metabolism dysfunction. Heme oxygenase-1 was indicated as a candidate biomarker for ACR (63). Another study found 41 differential expressed proteins in sera of ACR patients, including complement component 4q and 1q that could also predict ACR (64).

BUP studies in lung transplantation focused on determining biomarkers and mechanisms of chronic lung allograft dysfunction (CLAD), a major contributor to poor long-term transplant outcomes. BAL fluid analysis showed that the ratio of Clara cell protein to lysozyme has diagnostic power (65). In addition, the analysis of BAL from CLAD patients and controls by parallel reaction monitoring (PRM) assays targeting Angiotensin II-regulated proteins showed that these assays could diagnose patients with graft inflammation and predict chronic graft dysfunction (66).

In heart transplantation, BUP sought to identify potential diagnostic and prognostic biomarkers of AR and graft dysfunction in blood. It was found that (1) a set of exosomal serum proteins could distinguish acute ACR and ABMR from no rejection patients (67); (2) BD alters plasma proteome causing alterations in several cellular processes, including coagulation and gluconeogenesis among others; (3) expression levels of lysine-specific demethylase 3A and Kallikrein (KLKB1) could predict graft dysfunction, and those of myosin Va and proteasome activator subunit 2 acute rejection (68). The potential outcome predictive value of KLKB1 was also suggested in serum macrovesicles by others (69).

## 3. Top-down proteomics in transplantation: the move from protein to proteoform

Protein complexity is much broader than the amino acid sequence determined by the genetic code. A single gene can produce different forms of proteins called “proteoforms” deriving from genetic variation, alternative splicing, and PTMs (70). Different proteoforms can have different functions and contribute to different phenotypes. Therefore, they can be more accurate biomarkers of health and disease statuses and therapeutic targets than proteins and their identification advance SOT research (71, 72).

Several examples corroborate this hypothesis: (1) proteoform-level information of histones, which are DNA binding proteins regulating gene expression through combinations of PTMs (73), correlate better to the biological outcome than the overall abundance of histones or individual sites (74–77); (2) proteoforms better distinguish cell types than proteins in PBMCs (78); (3) serum apolipoproteins A-I and A-II proteoforms specifically associate with cardiometabolic indices while no significant differences were detected at protein level (79); and (4) proteoforms of KRAS have distinct signaling functions in colorectal cancer (80).

TDP is able to thoroughly characterize proteoforms informing on exact mass, unambiguous isoform assignment, abundance, and high-value PTM information (i.e., stoichiometry, types, and sites). While BUP identifies and quantifies protein groups with great sensitivity and dynamic range, it has a big limitation in PTMs and proteoform identification due to the inaccurate “inference” of proteoform from peptide-level data (81). An example is illustrated in Figure 2A. If a hypothetical protein has three phosphorylation sites on different tryptic peptides, BUP can neither determine the proteoform composition of the original protein group, whether all three phosphorylations occur together on one proteoform or a mixture of mono- to triple-phosphorylated proteoforms or quantify their stoichiometries. TDP overcomes this issue by analyzing intact proteins. In TDP, which is often coupled with LC, proteoforms are separated and directly ionized into mass spectrometers where the intact mass spectra (MS^1^) are collected, and ions further fragmented (MS^2^) for sequencing and PTM site identification (Figure 2B). Further advantages and limitations of TDP and BUP were reviewed elsewhere (20, 82).

TDP has been applied in transplantation studies aimed at identifying novel proteoform diagnostic and prognostic biomarkers of graft rejection and dysfunction. A TDP study conducted on PBMCs from 10 transplant recipients with and without acute rejection (AR) identified and quantified 3,000 unique proteoforms (83). Of these, 111 showed significant differential expression in the two conditions, including stress-associated ER protein 1 and 60S ribosomal protein L35a (84, 85).

Another study analyzed PBMCs from a small cohort of liver-transplanted patients to identify biomarkers of different transplant outcome: transplant excellence (TX), acute dysfunction with no rejection (ADNR), and AR. The analysis revealed 82 differentially expressed proteoforms and indicated that the most significant variations were associated with chemokine/cytokine signaling and cytoskeletal regulation (86).

In a follow-up “discovery mode” TDP study conducted on a larger single-center cohort (*n* = 75), 61 differentially expressed proteoforms were identified (78). Validation was conducted on patients from another multicenter cohort using a “targeted” method with narrower MS^1^ scan window focusing on the 61 proteoforms of interest. Among these proteoforms, 24 were confirmed to be biomarker candidates for liver-transplant dysfunction or rejection. For more stringent validation, Huang et al. recently described the proteoform reaction monitoring (PfRM) workflow, which resembles the BUP targeted MS^2^ quantification. In PfRM, the selected 24 proteoforms were fragmented and the resulted ions used for a more sensitive and specific quantification to femtomolar magnitude (87). We anticipate that future studies will likely validate these proteoforms on a larger scale.

TDP was also used in heart allograft evaluation studies. Zhang et al. identified the phosphorylation of cardiac troponin I (cTnI) as a candidate biomarker for chronic heart failure (CHF). Specifically, they found that the relative abundances of total phosphorylated cTnI forms in postmortem heart tissues decreased from those with normal cardiac function to end-stage CHF. Similar studies conducted on heart transplant tissues revealed a significant loss of phosphorylation on cTnI Ser22/23 sites in end-stage failing hearts compared to those from non-failing donors (88). Profiling of other proteoform targets in heart tissues are currently ongoing (89–91). Notably, while only a few studies applied TDP to transplant research, TDP has shown promising translational values in other clinical areas (92) and is expected to make a similar impact in the transplantation field in the future (93).

TDP has several technical challenges that need to be overcome to reach its full potential. One challenge is associated with limitations in the size of the protein analytes due to difficulties in separation (94, 95). Drown et al. recently demonstrated that coupling capillary zone electrophoresis (CZE) with TDP further increases proteoform identification by 1.7-fold across five different tissue types compared to using LCMS only (96). This technological improvement could be applied to graft evaluation in the future. Another TDP challenge is the overlapping of different species, charge states and isotopic distributions in the limited *m/z* space of mass spectra. To address this, Kafader et al. developed Individual Ion Mass Spectrometry (I^2^MS) that was recently used for analyzing antibody repertoires against SARS-CoV-2 in COVID-19 patients and vaccinated individuals (97–99).

## 4. Next frontiers in proteomics for advancing transplant outcomes

MS-based proteomics is a promising approach for SOT biomarker and therapeutic target discovery by providing global profiling and quantitative readout of proteoform changes (20, 100). However, current proteomic workflows in SOT research focus on the analysis of bulk clinical samples. Those fall short in reporting proteomic changes in specific cell types and populations and lose information on the spatial origin of the cells in solid tissues.

Protein distributions and changes in tissues and cells have been traditionally probed by antibody-based approaches in SOT research (101–103). These studies have been focused on proteins with prior knowledge and have limitations in the discovery of novel protein-based biomarkers. In recent years, emerging spatial and single cell proteomics technologies have enabled the discovery of protein signatures specific to functional tissue units, cellular neighborhoods, and cell types. As infiltrating immune cell populations are heterogeneous in allograft rejection, single cell-resolved proteome measurement is crucial to elucidate transplant rejection pathology and targeted cell therapy (104). BUP-based single cell proteomic (SCP) technologies such as SCoPE-MS and NANOPOTS have enabled the characterization of single cells in heterogeneous cell populations (105, 106). These techniques emerged from advances in high-throughput cell isolation, preparation, and automated small volume cell processing. By interfacing isobaric tag-based high-sensitivity LC-MS workflows, thousands of proteins from single cells were quantified, opening new avenues for SOT protein biomarker discovery (107–114) (Figure 2A). In one example applied to organ transplantation research, despite not single cells, Clotet-Freixas et al. collected glomerular and tubulointerstitial compartments of transplant kidney biopsies using laser capture microdissection that identified protein signatures responsible for extracellular matrix remodeling in antibody-mediated kidney allograft rejection (115). However, despite significant advances in SCP, current technologies have limitations in experimental throughput and rare cell profiling, and proteoform-level measurement is yet to be achieved.

Spatial mapping of proteins directly in SOT biopsies preserves the origin of cells of interest and their surrounding microenvironment (116). Immunostaining-based multiplexed protein imaging techniques have enabled dozens of proteins to be imaged simultaneously in a tissue section. For example, Imaging Mass Cytometry (IMC) (117) has been applied to kidney imaging and discovered spatial heterogeneity of immune cells in cortex and medulla and a rare subset of proximal tubule cell population representing regenerating cells (118). Despite significant advances in the number of imaged proteins, these techniques rely on pre-defined antibody panels for proteins with prior knowledge. Imaging multiple protein markers in one tissue sample in solid organ transplant biopsies significantly benefits SOT research where availability and quantity of tissue biopsies are extremely limited. Recently, protein and proteoform imaging has been enabled by spatially resolved MS imaging (MSI) approaches. Aside from MSI workflows utilizing spatial tissue sampling coupled to LC-MS BUP proteomics (119), laser and liquid extraction-based scanning MS imaging probes have enabled spatial mapping of dozens to hundreds of intact proteins and proteoforms (120–122). Specifically, liquid sampling probes (nano-DESI) coupled with single molecule MS detection have allowed imaging of intact proteoforms and native protein complexes directly from tissue sections in a recently introduced technique called proteoform imaging MS (PiMS) (114, 123–127) (Figure 2C). In the first report, PiMS detected ∼400 proteoforms from healthy human kidney tissues with many of them showing localizations to different kidney tissue compartments and cellular neighborhoods. These techniques can be readily adapted to tissue biopsies in SOT research for protein and proteoform biomarker discovery that were previously obscured by bulk measurements.

## 5. Conclusion

Although several promising biomarkers of graft injury and rejection have been identified (14, 128, 129), robust diagnostic and prognostic are still unavailable. In addition, there are no efficacious treatments to block injury and rejection.

Proteins are the direct drivers of biological functions and phenotypes, therefore, represent ideal biomarkers and druggable targets. New advances in MS-based proteomics have enabled the analysis of proteomes at unprecedented levels of granularity by increasing the proteome coverage and providing information at proteoform, single-cell and spatial resolution. Such information could elucidate mechanisms underlying injury and rejection and lead to novel biomarkers and therapeutic strategies. However, technical, and economic challenges still prevent the transition of these new technologies from discovery to clinical applications (20, 93, 130).

Finally, the adoption of a multi-omics approach integrating data generated by those powerful proteomics technologies, genomics, transcriptomics, and metabolomics will help to unveil mechanisms linking genotype and phenotype in SOT and drive the identification of multi-biomolecules biomarker signatures, the development of new therapeutics, and the advancement of precise medicine (131).
